# A Quality Improvement Project: Providing Music Therapy for Premature Infants in the Neonatal Intensive Care Unit

**DOI:** 10.1097/pq9.0000000000000856

**Published:** 2025-12-11

**Authors:** Supriya Bisht, Lan Jiang, Melissa Frascone, Ellen Shatzkin, Kristen Buck, Katie R Forman

**Affiliations:** From the * Division of Neonatology, Department of Pediatrics, The Children’s Hospital at Montefiore, Bronx, N.Y.; † Department of Pediatrics, The Children’s Hospital at Montefiore, Bronx, N.Y.

## Abstract

**Introduction:**

Preterm infants require adequate positive auditory stimulation for their development. Music therapy (MT) offers several benefits. Recorded music is recommended when live music is unavailable. This quality improvement project aimed to provide recorded MT to preterm infants.

**Methods:**

The team led this quality improvement project in a level IV neonatal intensive care unit from December 2022 to April 2024, with the aim that within 6 months of starting it, 50% of preterm infants born at a gestational age of less than 32 weeks who met physiological stability criteria would receive MT sessions. The Plan-Do-Study-Act model included interventions such as educating neonatal intensive care unit staff and providers about MT, creating memory aids, and streamlining documentation. Recorded Mozart music was played through bedside speakers 3 times a week for 30-minute sessions. The outcome measure tracked the percentage of eligible infants who received MT. Process measures evaluated the percentage of MT sessions provided. Balancing measures included the termination of MT due to clinical instability.

**Results:**

Eighty-seven infants received MT. The mean birth gestational age of the infants was 28.4 weeks. MT was initiated at a mean postmenstrual age of 33.4 weeks and continued until a mean age of 39.1 weeks. The percentage of infants receiving MT increased from 50% in the first month to 100% and stayed at a mean of 94.2%. The number of eligible MT sessions received per infant was 41.9% (average of 6.7 sessions). A total of 76.7% of nurses identified no barriers, and parents reported satisfaction with the MT program.

**Conclusions:**

Recorded MT can be effectively provided. Multidisciplinary teamwork is required to sustain efforts in providing MT sessions.

## INTRODUCTION

A critical phase of neonatal brain growth and development occurs during the last trimester. Preterm birth negatively affects the normal brain maturation process. The auditory system in the developing fetus is formed by around 20 weeks of gestational age (GA).^1^ The fetus can respond to sound by approximately 25–27 weeks of GA.^2^ Amniotic fluid and maternal tissues act as a physical barrier to high-frequency sounds, allowing low-volume, low-frequency sounds to filter through and appropriately stimulate the developing auditory system of the fetus in utero.^3^ The neonatal intensive care unit (NICU) environment is stressful for preterm infants and filled with loud, unpredictable, high-pitched sounds that mismatch their physiological needs. Although unwanted noises and overstimulation in the NICU are undesirable, it is equally necessary to provide adequate positive auditory stimulation for appropriate development. Music therapy (MT) helps bridge this gap.^2,4^

Music helps decrease heart rates, stress, and salivary cortisol levels in preterm infants.^5^ Additionally, MT improves nonnutritive sucking, oxygen saturation, and weight gain, thereby reducing the overall duration of the NICU stay.^5,6^ MT also enhances the motor development in postterm infants,^7^ causing a positive effect on both physiological and behavioral parameters of infants.^4^

Sa de Almeida et al^8^ conducted a randomized controlled study on infants (GA <32 weeks at birth) and exposed them to recorded MT. A subsequent multimodal brain magnetic resonance imaging at term suggested that MT has a positive effect on the maturation of auditory and emotional processing pathways.^8^ Loewy et al^9^ provided different types of auditory stimulation, including lullabies and sounds that simulated the womb or maternal heartbeat, and demonstrated that all had varying positive effects on the preterm infants. Exposure to appropriate music for preterm infants has also been shown to help reduce parental stress.^9,10^

In developing an MT program for the NICU, the type, volume, and duration of music must be considered. Although live music has more advantages than recorded music,^3,11,12^ the use of recorded music has had a positive impact on infants.^6,13–15^ Recorded music can be safely administered to stable preterm infants with 28 weeks postmenstrual age (PMA).^16^ The benefits are significant for preterm infants between 28 and 35 weeks PMA.^14^ Multiple studies have provided music for durations ranging between 20 and 45 minutes,^3,11,17^ with music sessions varying from 3 times a week to daily sessions.^3,9^

Research in children and adults has shown a link between exposure to Mozart's music and enhanced cognition, reduced pain, and improved mood,^18^ phenomenon termed the “Mozart effect.” In neonates, studies suggest that exposure to Mozart music, compared with other types of music, reduces resting energy expenditure^5,19^ and is used in many MT studies due to its positive effect on physiological parameters.^17,20^ Most studies using recorded music have used speakers rather than headphones,^21^ with the music volume ranging from 55^11^ to −70 dB.^3^ In contrast, some studies suggest maintaining a volume of 65–75 dB is appropriate for neonates.^14,17^

Meta-analysis indicates that well-designed implementation of recorded MT is a safe and efficient intervention to improve infant outcomes.^3^ MT in the NICU occurs at the bedside, promoting parental bonding and enabling parents to provide positive stimulation to their infants. It is well received by parents who appreciate the opportunity to be involved in their infant care.^15^

We aimed to provide MT to the preterm infants in our NICU. This quality improvement (QI) initiative was launched with a specific, measurable, achievable, realistic, and timely (SMART) aim that within 6 months of starting the QI project (June 2023), 50% of admitted preterm infants born at greater than 32 weeks of GA, meeting physiological stability criteria, would receive MT sessions during their hospitalization.

## METHODS

The QI was conducted in a level IV NICU at a New York State regional perinatal center in Bronx County, a major regional perinatal center with an annual admission rate of 600 infants, with approximately 100 of whom were born at less than 32 weeks GA. At any given time, 10–12 newborns met the eligibility criteria. The NICU is an open-bay unit. Critically ill and unstable infants are cohorted in 1 room, 2 rooms are dedicated for convalescing infants, and 1 room is for infants in the stable feeding phase.

NICU staff, advanced practice providers, fellows, and residents were educated about the MT program, and the QI team conducted comprehensive training for the NICU staff to use the MT instruments. Recorded Mozart music was played through a speaker 3 times a week during the day shift, with designated MT days (Monday, Wednesday, and Friday) in the unit. A 30-minute MT session was conducted at a volume of 65–70 dB. A sound meter was used to monitor the music volume. The speakers were positioned at a suitable distance from the infant (30 cm from the ears).^17,19^ The MT speaker was kept at the bedside of each participant until discharge or transfer.

### Ethical Considerations

The Albert Einstein College of Medicine Network Performance Group registered the QI initiative.

### Study Population and Study Period

Eligibility criteria included preterm infants born at a GA of less than 32 weeks who were hemodynamically stable, with respiratory support of 30% FiO2 or less and +6 cm H20 or less of continuous positive airway pressure, and were on full enteral feeds. Infants born at a GA of 32 weeks or more, those who were hemodynamically unstable, received higher levels of respiratory support, or had severe congenital anomalies were excluded from the QI. Once infant enrollment was completed for MT, the infants continued to receive MT, even if their respiratory support or feeding pattern changed, provided they remained hemodynamically stable. Parents were provided with an information sheet at the start of their infant’s NICU admission to explain the MT project and were also informed about the project again at the start of MT sessions. They had the opportunity to decline participation.

### Planning the Intervention

The multidisciplinary QI team comprised nurses, advanced practice providers, physicians (including trainees), and an NICU feeding therapist. A key driver diagram (Fig. 1) and an Ishikawa cause-and-effect diagram (Fig. 2) identified the important drivers and limitations to implement MT. The team identified 4 primary drivers for this QI: improving NICU care, NICU staff involvement, family satisfaction, and creating standardized processes. The QI team selected appropriate Mozart symphonies and purchased the appropriate MT speakers and sound meters. The Plan-Do-Study-Act (PDSA) model was used to gather feedback and facilitate improvement. The QI team collected the data and analyzed it using run and statistical process control (SPC) charts, and met at regular intervals to discuss improvements.

**Fig. 1.**
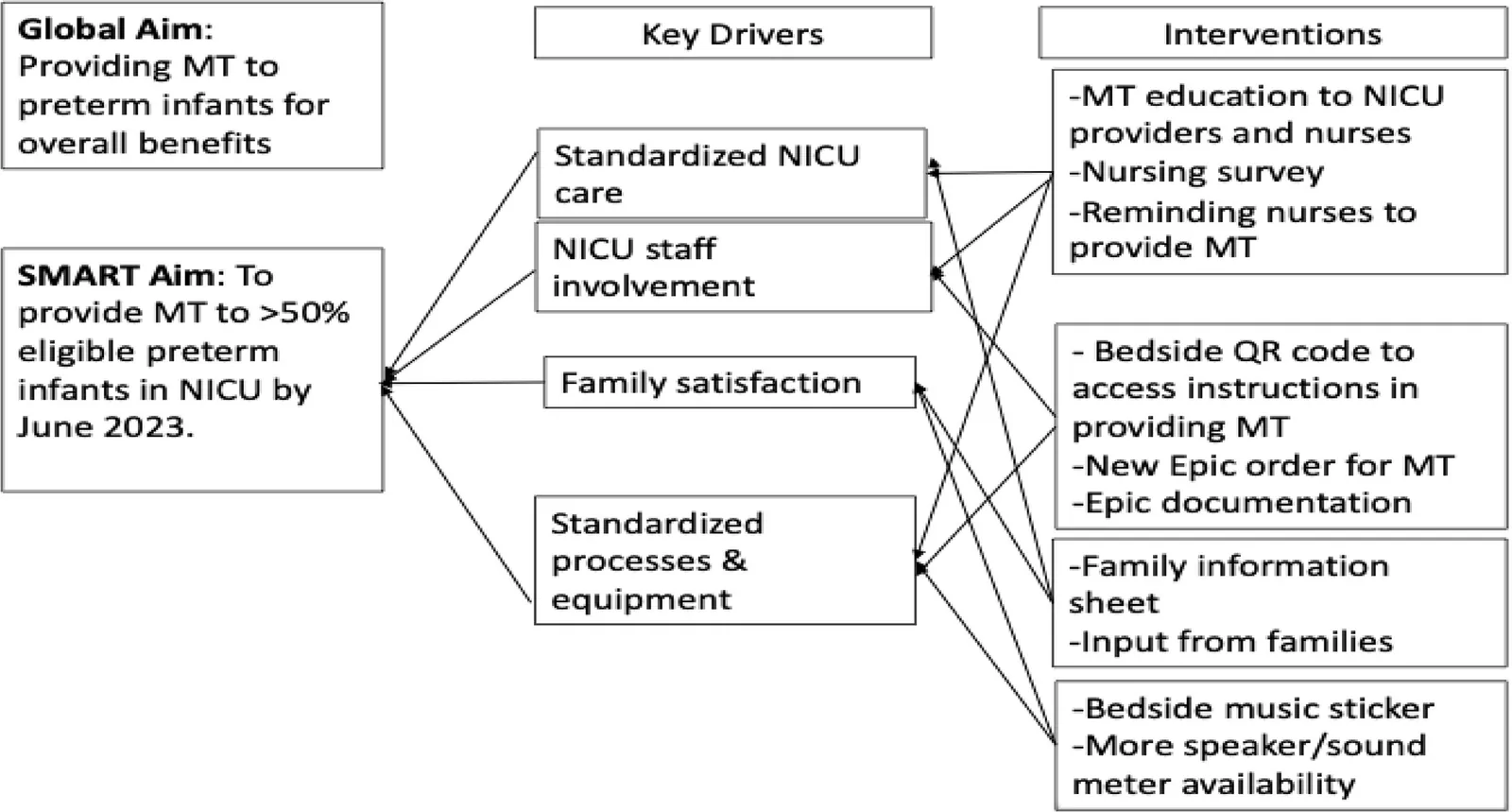
Key driver diagram.

**Fig. 2.**
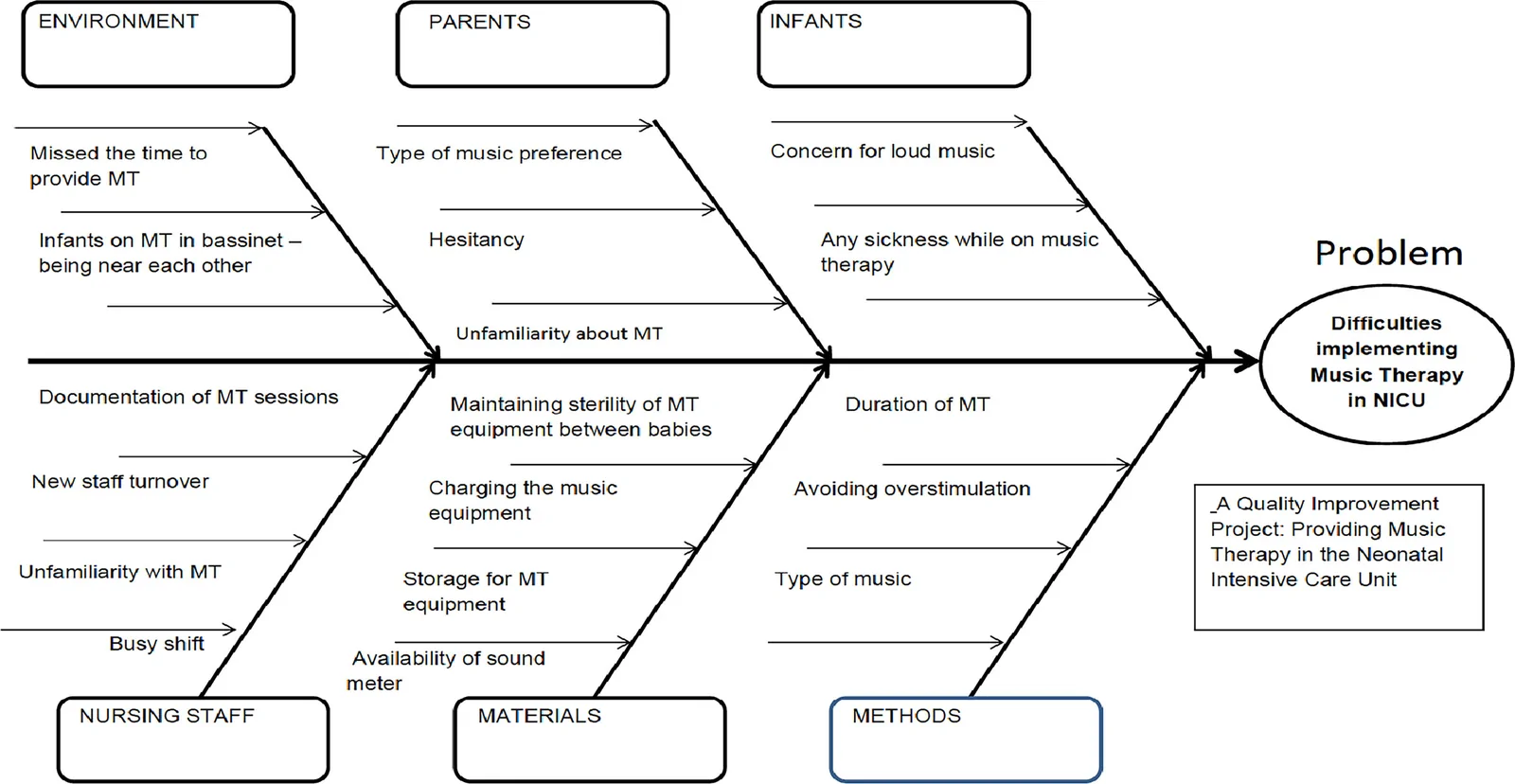
Fishbone diagram for MT.

### PDSA Cycle 1

The team implemented a change package including 4 interventions during PDSA 1 (December 2022 to March 2023):

1. The entire NICU staff received education about the benefits of MT, and the new proposed workflow and process were explained in a short slide presentation.
2. Newly enrolled infants were highlighted during the daily morning multidisciplinary team huddle to promote awareness.
3. Key stakeholders messaged the rounding NICU care team via a secure chat platform on MT days to remind them about enrolled babies and request a discussion during rounds with the nurse that day.
4. An MT sticker was created as a visual reminder tool that was placed at the patient’s bedside to remind the nurses and care team that the infant was enrolled to receive MT.

### PDSA Cycle 2

The change packet for PDSA cycle 2 (April 2023 to July 2023) included the addition of 2 interventions and 1 project assessment:

1. The QI team created a new quick response (QR) code in the MT sticker that linked to information and a short video about how to provide MT.
2. Key stakeholders began sending reminders to the assigned nurses as well on MT days via a secure chat application.
3. The team surveyed NICU providers and nurses with a short questionnaire to assess their knowledge, concerns, and perceived barriers.

### PDSA Cycle 3

Two new interventions were implemented (August 2023 to November 2023):

1. A standardized order was added in the electronic medical record (EMR) to be placed on infants who qualified for MT. Our NICU unit used Epic as the EMR (Epic Systems, Verona, WI). Once placed, this order prompted a formal “nursing task” within the EMR to provide MT during their shift on identified MT days. This task linked the updated MT flowsheet rows within the EMR, which were more user-friendly and included a place to document the tolerance of MT.
2. Given improved compliance, the team purchased additional music speakers.

### PDSA Cycle 4

During the fourth PDSA cycle (December 2023 to February 2024), the team focused on expanding resident education about the MT project. A resident stakeholder implemented peer-to-peer education modules for the new residents. Additionally, there was a transition in electronic secure chat platforms at the institutional level. The new platform was integrated into the EMR, and its uptake and use were nearly universal among providers and nurses. The QI team also surveyed parents to assess their perspective on MT and gather feedback.

### Sustainment Phase

During the sustainment phase (starting after March 2024, when our primary outcome was consistently stable), the QI team continued the interventions performed from the prior PDSA cycles to provide MT, ensuring compliance with the provision of MT for all qualifying infants.

### Study Measures and Data Collection

The primary outcome measure tracked the percentage of eligible infants who successfully received MT for a minimum of 1 session. The process measure monitored compliance by determining the percentage of completed MT sessions provided to each infant per eligible sessions each month. Balancing measures included documenting premature termination of MT or signs of overstimulation (clinical instability), such as apnea, bradycardia, desaturation, vomiting, or any other concerning clinical changes during MT. The QI team also collected demographic data on infants receiving MT, including GA, birth weight, sex, PMA, and growth parameters.

This initiative was the first time that an MT program was formally integrated into the NICU care. Although nurses occasionally exposed some older infants to music at their discretion, this was not standardized, so baseline data for our primary outcome measure did not exist.

### Data Analysis

The QI team developed real-time run charts and SPC charts using QI Macros for Excel (KnowWare International Inc., Denver, CO) to evaluate the data. For SPC charts, the control limits were the upper control limit) and lower control limit, which were set at 3 SDs above and below the center line, respectively. Eight consecutive data points above the mean were considered indicative of special cause variation.

## RESULTS

Eighty-seven eligible infants received MT from December 2022 to April 2024. The average PMA at the start of MT was 33.4 weeks, and infants received MT until the mean PMA of 39.1 weeks. The youngest preterm infant was born at 22.5 weeks of GA and received MT starting at 35 weeks of PMA. The demographic details of the infants are presented in Supplemental Digital Content 1. (**See table 1, Supplemental Digital Content 1**, which displays the demographic data of infants receiving MT, https://links.lww.com/PQ9/A722).

Most infants were started on MT with continuous positive airway pressure support (n = 48, 55.6%), followed by nasal cannula/high-flow nasal cannula (n = 22, 25.3%).

The percentage of eligible infants who received at least 1 MT session was 50% in the first month. With the broader implementation of the PDSA 1 change package, the mean increased to 88.1%. In March 2023, there was a special cause variation, and the mean increased to 94.2%, as shown in the SPC-P chart (Fig. 3).

**Fig. 3.**
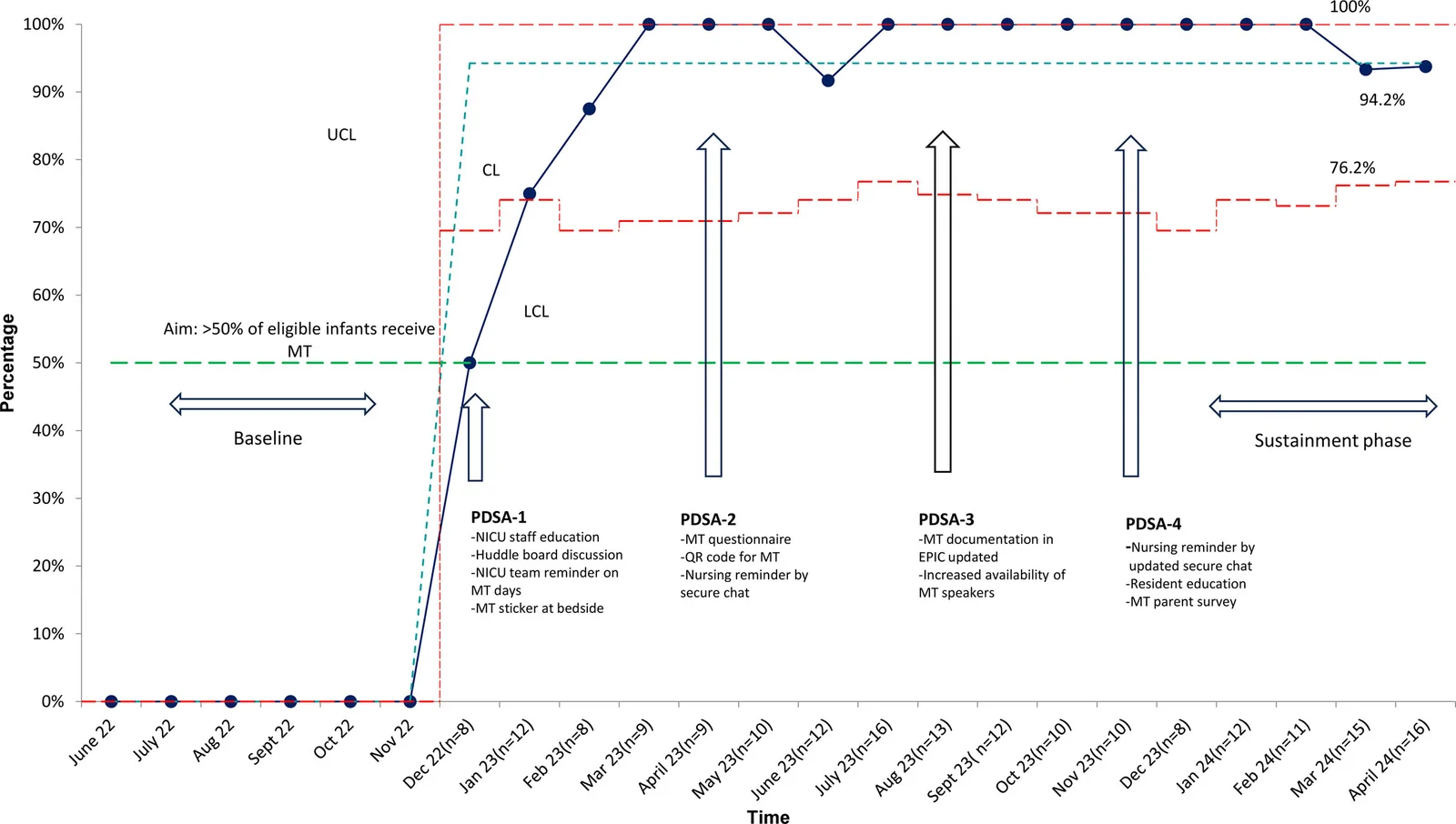
Percentage of eligible infants receiving any MT: P chart. CL, control limit; LCL, lower control limit; UCL, upper control limit.

The process measure for compliance is shown in the SPC-P chart (Fig. 4). The average percentage of eligible MT sessions received per infant was 41.9% (with an average of 6.7 sessions, Figure 5). Thirty-seven infants received up to 5 sessions, whereas the remaining infants received more than 6 sessions during their NICU stay and enrollment. Two infants received around 22 sessions, and no particular reason for the difference in MT sessions among the infants was observed.

**Fig. 4.**
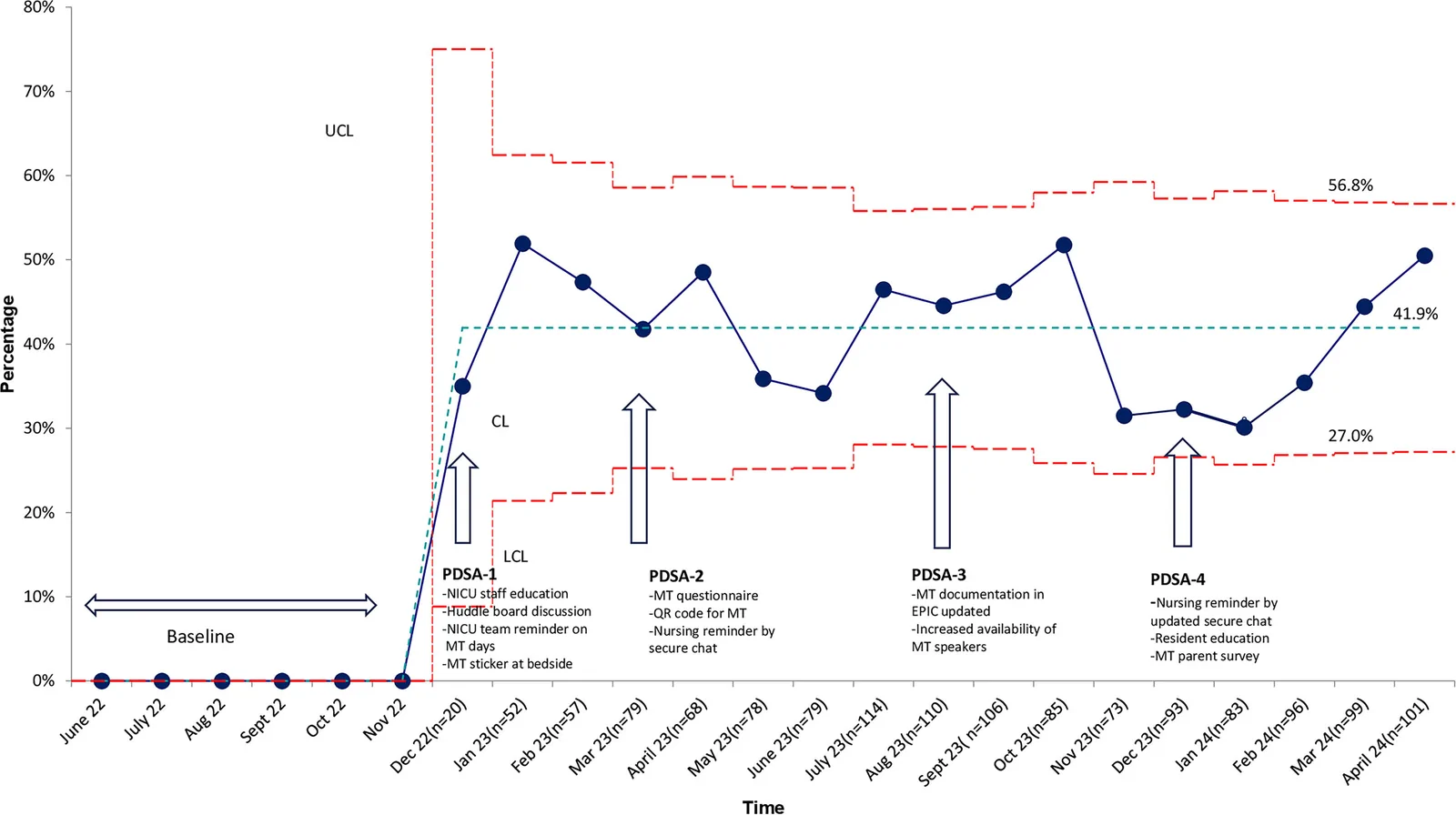
Eligible MT sessions completed: P chart. CL, control limit; LCL, lower control limit UCL, upper control limit.

**Fig. 5.**
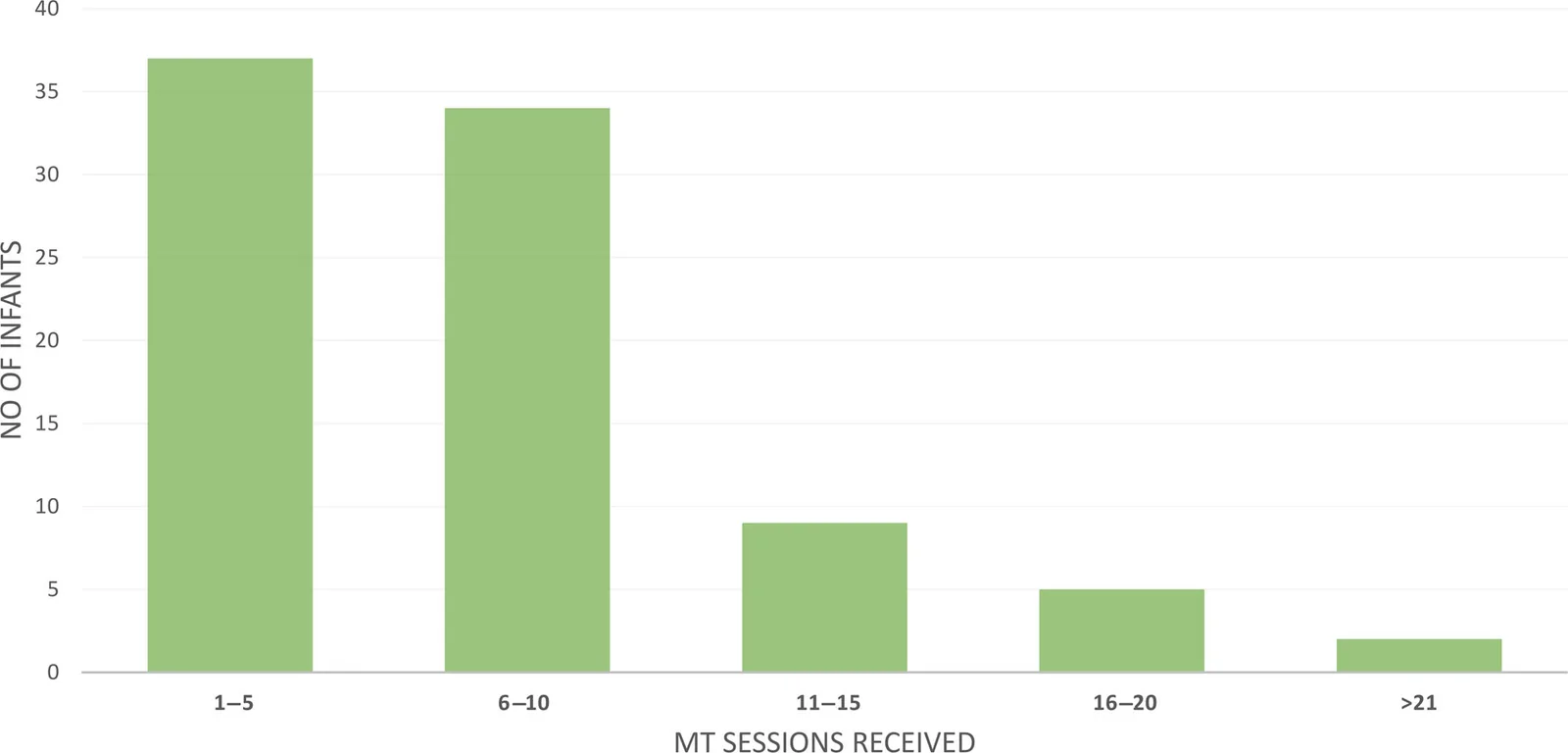
MT sessions received during NICU stay.

Balancing measures indicated that the infants remained stable during MT, with no documented overstimulation, apnea, or desaturation events during the provided MT sessions.

Nursing responses (Table 1) to the assessment survey showed that 76.7% of nurses identified no barriers to providing and documenting MT, and 91% felt that music had a positive impact on the infants. A total of 13% of nurses indicated that documentation of providing MT was missed, despite providing the MT sessions. Common barriers to MT included busy shifts, unfamiliarity with using MT instruments, and misplaced MT instruments.

**Table 1.** Survey of Nurses and Parents

Nurses Survey: Barriers for MT		Parents Survey: Benefits of MT	
No barriers	76.7%	Infant more calm	64.3%
Forgot documentation	13.0%	Increased bonding	14.3%
Busy shift	7.0%	Infant sleeps well	7.1%
Don’t know how to provide/document MT or misplaced instruments	3.3%	Cannot comment	14.3%

The parent survey (Table 1) indicated that 71.4% preferred Mozart music, whereas the remainder had no specific music preference. The majority (85.7%) of the parents perceived that their infant was calmer during MT, took more feeds, or had increased parent–infant bonding. The remaining parents did not interact with the baby during the MT session.

## DISCUSSION

The positive effects of providing MT to premature neonates are well described. In the quest to optimize neonatal neurodevelopment and enhance parental experiences in the NICU, therapeutic interventions are prioritized nationwide to reinforce these goals. Although the benefits of live music are well known,^11^ it can be expensive and challenging to find and engage board-certified music therapists, particularly in low-resource environments. There is growing evidence that exposure to recorded music is also beneficial^9^; however, there are currently no standardized recommendations about how and when to integrate recorded music into neonatal care. Studies have provided MT to a variable age range, involving infants born preterm at 25 weeks GA to term infants.^3^ Nursing literature reports a preference for recorded music.^22,23^ The American Academy of Pediatrics (AAP) guidelines address general noise exposure.^24^ These guidelines adopt the US Environmental Protection Agency’s (1974)^25^ recommendations, which state that the NICU noise level should remain approximately 45 dBA. and the noise should not exceed 70 dBA.^26^ This discussion did not differentiate between sound levels for positive auditory stimulation versus ambient noise levels. A variety of studies have since provided MT at a volume on the upper end of what is recommended by the American Academy of Pediatrics with success.^3^ Our QI team provided a sound meter in every room to monitor the sound level of the music.^27^ To our knowledge, this is the first published QI that demonstrates the feasibility of incorporating recorded MT in the NICU.

The QI team focused on the methodologies for QI compliance studies and created a key driver diagram (Fig. 1) and an Ishikawa cause-and-effect diagram (Fig. 2) for the MT program. Interventions included MT education, daily discussions on MT eligibility during huddles and rounds, monthly nursing feedback, and the placement of bedside MT stickers, which were modified to include a QR code linking information about MT. Additionally, the EMR was modified to include MT orders, new nursing flow sheet rows, and new nursing task activities that served as digital memory aids.

This QI project was the first time that MT was implemented in the NICU in a standardized manner; therefore, the baseline data were 0. After implementing change package no. 1, there was rapid adoption, and the QI team achieved the SMART aim within the first PDSA cycle (Fig. 3). Specifically, 50% of eligible infants received at least 1 session of MT, which continued to improve and ultimately reached 100%. There was a slight, acceptable drop in March and April 2024. During that time, many infants were eligible for MT; however, a limitation in the number of speakers and supplies to meet the need decreased the average.

Despite the early success in achieving our SMART aim, there was a wide variability in compliance with MT (Fig. 4). All infants received at least 1 session after enrollment in the QI; however, none of the patients received all the documented eligible sessions. MT session compliance rates dropped during times of high nursing turnover (May–June 2023 and November–February 2024). In a nursing assessment conducted in July 2023, 13% of nurses reported that, despite providing MT, its documentation in the EMR was often missed. This observation suggests that the actual number of MT sessions received was higher than the reported mean of 41.3% (with an average of 6.7 sessions received). The barriers identified by nurses were busy shifts, a lack of knowledge about how to use an MT speaker/sound meter, and misplaced or noncharged speakers. The increased frequency of nursing training and the incorporation of QR code–linked training materials improved compliance. Both nurses’ and parents’ feedback regarding patient experience was positive, which aligns with the existing literature.^22,23,27^ Only 1 family declined to participate in the MT initiative. Our parental survey results showed that 85.7% of the parents felt that their child had visible improvement while receiving MT. Kobus et al^28^ showed similar numbers with 94% of parents of infants born at greater than 32 weeks GA observing positive changes in their infant during MT.

Before starting MT, the mean weight percentile of the infants in our QI initiative was 22.0, whereas at the time of MT discontinuation at discharge/transfer, the weight percentile was 26.1. Few studies have mentioned that music improves weight gain in infants.^5,6^ However, a recent study found no difference in weight parameters in infants receiving MT, with prematurity being the most important factor affecting weight.^29^ The overall positive effects of music on neonates^30^ favor MT. The weight percentiles of the infants improved in our QI initiative. At the same time, it is desirable to aim for the maximum number of complete MT sessions to achieve associated benefits.

Although this project was received favorably and demonstrated the feasibility of implementing an MT program, several challenges remained. Recorded music requires resources to buy safe MT equipment. To maintain infection control, 1 device was assigned to each infant throughout their stay and was cleaned appropriately between uses. Perceived safety concerns were a postulated barrier to initiating this project; however, the early and rapid uptake of MT, along with minimal parent refusal, indicates that this concern was unfounded. The effect of staffing limitations, including high nursing ratios, novice nurses, and nursing fatigue, was mitigated by recurrent and easily accessible training and education. The open-bay layout of the NICU itself was a limitation, as there was no way to completely control residual noise from MT between patients. However, the infants’ beds were already separated by a much larger distance. However, this factor should be addressed if high- and low-acuity patients are situated in close proximity before the implementation of MT.

The QI team achieved sustainability through champions dedicated to continuing the established processes, providing nursing reeducation, and maintaining the MT equipment. As with any teaching institution, changes in trainee staffing limit the institutional memory. To optimize the maintenance phase, an advanced practice provider spearheaded the MT effort and guided new learners through the process.

## CONCLUSIONS

NICUs can successfully implement recorded MT for the qualifying infants. Continuous multidisciplinary team engagement is necessary to sustain efforts in providing MT. MT is perceived positively by the NICU staff and parents for its overall benefits. In the future, this group aims to provide MT to younger preterm infants and term infants experiencing painful procedures, and to include a recorded maternal singing voice as an alternative option. This QI initiative can serve as a model for other NICUs to develop an MT protocol, providing evidence-based, family-centered interventions for preterm infants.

## Supplementary Material


