# Supplementary material for: A Quality Improvement Project: Providing Music Therapy for Premature Infants in the Neonatal Intensive Care Unit
Source: Pediatr Qual Saf. 2025 Dec 11;10(6):e856. doi: 10.1097/pq9.0000000000000856 (PMC13619241; doi:10.1097/pq9.0000000000000856)
Supplement: Supplementary file 1 [file pqs-10-e856-s001.pdf]

Supplemental digital content 1(Table 1. Demographic data of infants on MT)

**Variables**	**Total N = 87**
**Birth Gestational Age (weeks)**	
Mean (±SD).	28.4 (±2.3)
**Race**	
White	7 (8%)
Black	34 (39%)
Other	46 (53%)
**Gender**	
Male	56(64.3%)
**Ethnicity**	
Non hispanic	47 (54%)
**Birthweight (grams)**	
Mean (±SD)	1082.1 (±339.4)
**Birth weight percentile (%)**	
Mean (±SD)	39.3 (±25.2)
**PMA at start of MT (weeks)**	
Mean (±SD)	33.4 (±2.5)
**Weight at start of MT (grams)**	
Mean (±SD)	1738.5 (±531.3)
**Weight centile at start of MT (%)**	
Mean (±SD)	22.0 (19.9%)
**PMA at MT discontinuation (weeks)**	
Mean (±SD)	39.1 (±4.4)
**Weight at MT discontinuation (grams)**	
Mean (±SD)	2917.7 (±881.4)
**Weight percentile at MT discontinuation (%)**	
Mean (±SD)	26.1 (±21.2)
